# Alginate Adsorbent Immobilization Technique Promotes Biobutanol Production by *Clostridium acetobutylicum* Under Extreme Condition of High Concentration of Organic Solvent

**DOI:** 10.3389/fmicb.2018.01071

**Published:** 2018-05-25

**Authors:** Zhuoliang Ye, Jingyi Song, Enhao Zhu, Xin Song, Xiaohui Chen, Xiaoting Hong

**Affiliations:** ^1^School of Chemical Engineering, Fuzhou University, Fuzhou, China; ^2^National Engineering Research Center for Chemical Fertilizer Catalyst, Fuzhou University, Fuzhou, China; ^3^Key Laboratory of Recycling and Eco-treatment of Waste Biomass of Zhejiang Province, Zhejiang University of Science and Technology, Hangzhou, China

**Keywords:** biobutanol, high solvent concentration, alginate adsorbent, microstructure, *in situ* extraction, bioenergy

## Abstract

In Acetone-Butanol-Ethanol fermentation, bacteria should tolerate high concentrations of solvent products, which inhibit bacteria growth and limit further increase of solvents to more than 20 g/L. Moreover, this limited solvent concentration significantly increases the cost of solvent separation through traditional approaches. In this study, alginate adsorbent immobilization technique was successfully developed to assist *in situ* extraction using octanol which is effective in extracting butanol but presents strong toxic effect to bacteria. The adsorbent improved solvent tolerance of *Clostridium acetobutylicum* under extreme condition of high concentration of organic solvent. Using the developed technique, more than 42% of added bacteria can be adsorbed to the adsorbent. Surface area of the adsorbent was more than 10 times greater than sodium alginate. Scanning electron microscope image shows that an abundant amount of pore structure was successfully developed on adsorbents, promoting bacteria adsorption. In adsorbent assisted ABE fermentation, there was 21.64 g/L butanol in extracting layer compared to negligible butanol produced with only the extractant but without the adsorbent, for the reason that adsorbent can reduce damaging exposure of *C. acetobutylicum* to octanol. The strategy can improve total butanol production with respect to traditional culture approach by more than 2.5 fold and save energy for subsequent butanol recovery, which effects can potentially make the biobutanol production more economically practical.

## Introduction

Acetone-butanol-ethanol (ABE) fermentation is first developed by the chemist Chaim Weizmannan to produce acetone and *n*-butanol. Products from ABE fermentation are about six parts of butanol, three parts of acetone and one part of ethanol. Particularly, butanol has received growing attention from the renewable biofuel industries for advantages such as sustainability, easy distribution, lower solubility in water compared to ethanol biofuel and a high heating value with similar energy density to gasoline (Jin et al., 2011). It can be mixed with gasoline at any blend ratio and even be used in pure form as diesel fuel.

High cost of traditional feedstocks, low solvent concentrations, and high separation cost of butanol product are three key factors making the ABE process less economically feasible (Gu et al., 2011; Visioli et al., 2014). Utilization of macroalgae is an attractive way to reduce feedstock cost (Wargacki et al., 2012). Red macroalgae containing mainly agar and carrageenan has the most abundant species of macroalgae, but usually requires galactose utilization pathway. There are many previous reports demonstrated that *Clostridium acetobutylicum* (*C. acetobutylicum*) has the ability to utilize the galactose in red macroalgae substrates (Kim et al., 2013; Tang et al., 2017), making it possible to reduce substrate cost in the future for ABE fermentation. Compared to solvent producing *Clostridium*, a natural metabolic pathway for galactose is lacked in many other bacteria.

The solvent products were typically less than 20 g/L due to toxic effect to bacteria. Because product concentration is low, biobutanol was recovered by energy-intensive distillation process, using up to 220% of the energy content of butanol (Patraşcu et al., 2017). ABE fermentation was discontinued due to unfavorable economic conditions compared with petrochemical industry. If it can be coupled with extraction using simple settings, restart of ABE fermentation can become more attractive. Instead of engineering a strain that can tolerate high concentration of solvents, development of better downstream process is more practical.

**FIGURE 1:**
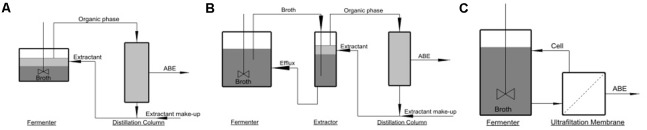
Schematic diagrams for **(A)** Extractive fermentation; **(B)** external solvent extraction; **(C)** perstraction.

Extracting techniques can be divided to *in situ* extraction (extractive fermentation), external solvent extraction, and membrane-assisted solvent extraction (perstraction). The three extracting techniques are schematized in **Figure 1**. In extractive fermentation (**Figure 1A**), desired product is extracted to organic phase (extractant) and then sent for enrichment in distillation column where extractant is recycled to the fermenter. Besides saving energy for butanol recovery, extractive fermentation can also reduce product inhibition and increase productivity. The extractant should be bio-compatible, and therefore were limited to a few kind of extractants such as oleyl alcohol and biodiesel (Yen and Wang, 2013). In external extraction (**Figure 1B**), the fermenter is integrated with an additional external extractor. The extractor can provide the product with fast mass transfer rate into organic phase. However, this technique requires a very large volume of extractant which increases the capital cost (Huang et al., 2014). Perstraction involves a membrane unit to continuously separate out desired products from broth, while the cells and other components are retained and recycled to the fermenter (**Figure 1C**). Perstraction allows use of the toxic extractant such as 1-dodecanol without direct contact to the cells (Tanaka et al., 2012), but durability of membrane is not well-reported. It is highly desirable to develop an *in situ* extraction technique that can use cheap and conventional extractants which have high partition coefficient of butanol.

It is proposed here that adsorbent immobilization technique can reduce damaging exposure of bacterium to toxic extractant. Octanol should have high partition coefficient for butanol, but there is seldom report of *in situ* extraction using *n*-octanol due to its toxicity (Groot et al., 1990). In this study, alginate adsorbent immobilization technique was developed to assist *in situ* extraction of butanol using *n*-octanol as extractant. The adsorbent was prepared by cross-linking alginate sodium with calcium chloride using cyclohexane as pore-forming agent. Nitrogen gas adsorption isotherms, scanning electron microscopy (SEM) imaging and infrared spectra (IR) were performed to study the interactions between the adsorbent and bacteria. Finally, fermentation and *in situ* extraction of butanol were performed using *C. acetobutylicum* immobilized by adsorbent, and compared to a counterpart without addition of adsorbent.

## Materials and Methods

### Materials

Calcium chloride, sodium alginate, and sodium chloride were purchased from Sinopharm Chemical Reagent, Co., Ltd. *Clostridium acetobutylicum* (CICC 8002) was obtained from China Center of Industrial Culture Collection (CICC).

### Preparation of the Adsorbents

The adsorbent was prepared by cross-linking alginate sodium (0.1 g) with 10 ml calcium chloride (5–10 g/L) using 10 ml cyclohexane to develop pore structures. Slurry was stirred vigorously at 800 rpm for 15 min. Resulting particles were precipitated, washed several times using 95% (v/v) ethanol and then air-dried for 2 days at room temperature in a laminar hood to prevent bacterial contamination.

The adsorbent particles were sieved in a set of United States standard sieves for 10 min to obtain mass distribution in following size ranges: >450, 300–450, 150–300, 105–150, 75–105, and <75 μm.

Binding of bacteria to the adsorbent was tested by adding bacteria in various concentrations to 0.1 g adsorbent and mixed for 1 h. Cell density of totally added bacteria and free bacteria after adsorption were determined by measuring light absorbency at a wavelength of 600 nm (Sarchami et al., 2016).

Fourier-transform infrared (FT-IR) imaging can obtain information on the chemical composition of biological sample and interactions between different components in a non-destructive way. In this study, IR spectra were obtained using a Nicolet iS50 attenuated total reflection (ATR) infrared spectrometer (Fisher Scientific, United States) in the range from 400 to 4000 cm^-1^ via ATR mode. The spectra were compared between adsorbent and adsorbent-bacteria samples to understand the type of interaction.

The morphology of *C. acetobutylicum* was recorded using a video microscope. The morphology of the adsorbent was examined by a scanning electron microscope operating at 5.0 kV (S-4800, Hitachi, Co., Japan).

Surface area was measured by nitrogen gas adsorption isotherms (Ye and Berson, 2014) using an adsorption apparatus (Micromeritics Instrument Corporation, ASAP 2020M) and calculated by means of the Brunauer–Emmett–Teller (BET) method. The cumulative volume of pores was determined using the Barrett–Joyner–Halenda (BJH) method.

The absorbent was evaluated using thermal gravimetric analysis (TGA; Netzsch STA 449C) under an Ar atmosphere flowing at a rate of 30 ml/min and at a heating rate of 10°C/min in the temperature range from 30 to 600°C. TGA provided information about thermal decomposition of the adsorbent sample. The first derivative of the TGA data (DTG data) was obtained from the measuring system, and provided peak temperatures of sample mass change.

### Fermentation

The medium was prepared as reported elsewhere (Ezeji and Blaschek, 2008), containing (unit in g/L) yeast extract 1, KH_2_PO_4_ 0.5, K_2_HPO_4_ 0.5, ammonium acetate 2.2, para-amino-benzoic acid 0.001, thiamine 0.001, biotin 0.00001, MgSO_4_⋅7H_2_O 0.2, MnSO_4_⋅7H_2_O 0.01, FeSO_4_⋅7H_2_O 0.01, NaCl 0.01, and glucose 50. In adsorbent assisted extractive fermentation, 13.3 g/L adsorbent was added in the medium. After autoclaved to achieve sterile condition, the bottles containing medium and adsorbent were purged with nitrogen to ensure anaerobic conditions. Total operating volume was 30 ml with 10% inoculum of *C. acetobutylicum.* Tests were run in 50 ml penicillin bottles and at 30 rpm using magnetic stirrer bar for mixing. 10 ml *n-*octanol was added above the medium after 12 h to extract solvent products. Improvement in butanol production by the adsorbent assisted extractive fermentation can be compared with respect to traditional culture approach without either adsorbent added at the beginning or extractant added after 12 h in the fermentation. Meanwhile, another control experiment was carried out without addition of adsorbent but with addition of extractant after 12-h fermentation, while other fermentation conditions are the same as listed in adsorbent assisted extractive fermentation. This control experiment can help understand how the adsorbent reduces toxic effect of octanol to bacteria.

Concentrations of acetone, butanol, and ethanol were determined via gas chromatography (GC) equipped with a GDX-103 column and a flame ionization detector (FID) using propanol as internal standard. The extraction performance of each product was evaluated taking into account the partition coefficient (*K*_p_). The partition coefficient was calculated by Equation (1):

(1) $$K_{p}\;=\;\frac{C_{org}}{C_{aq}}$$

where *C*_org_ and *C*_aq_ are the equilibrium concentrations in the organic and the aqueous phase, respectively.

## Results and Discussion

### Adsorption of Bacteria

**FIGURE 2:**
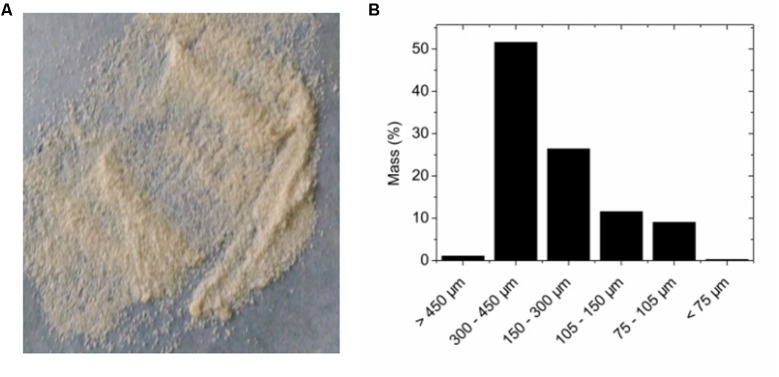
**(A)** Visual appearance of alginate-based adsorbent. **(B)** Particle size distribution of the adsorbent.

**FIGURE 3:**
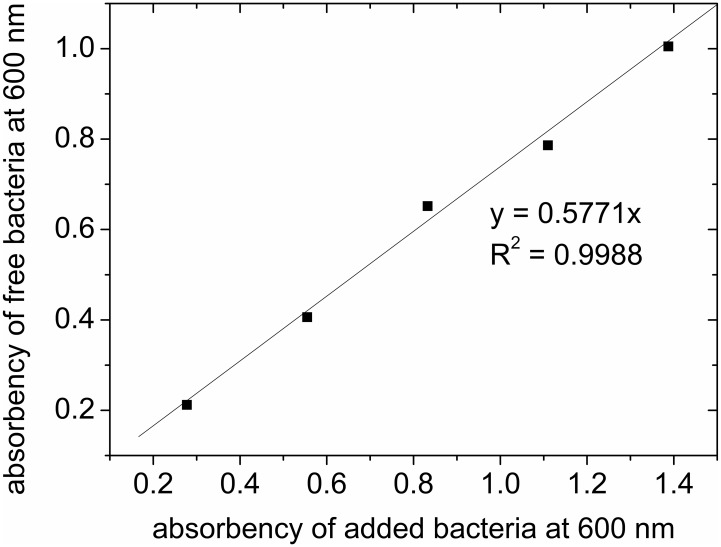
Binding of bacteria to the adsorbent.

The prepared adsorbents were in particle shape (**Figure 2A**), with sizes mostly below 450 μm (**Figure 2B**). The small size of the adsorbents allowed fast adsorption of bacteria. When bacteria binding to the adsorbent was tested, the adsorption data can be fitted with a linear function. The ratio of unbound bacteria to total bacteria was 0.5771, suggesting more than 42% of added bacteria can be successfully adsorbed to the adsorbents (**Figure 3**). The adsorption is expected to improve organic solvent tolerance of bacteria under the extreme condition of high concentration of organic solvent.

### Binding Mechanism

**FIGURE 4:**
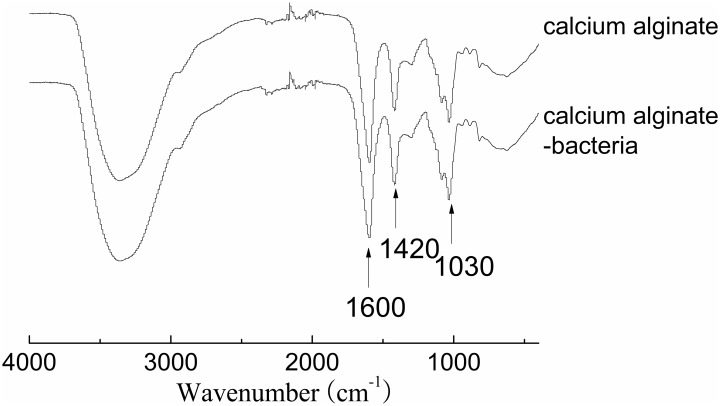
IR spectra of the alginate adsorbent and alginate-bacteria sample.

**FIGURE 5:**
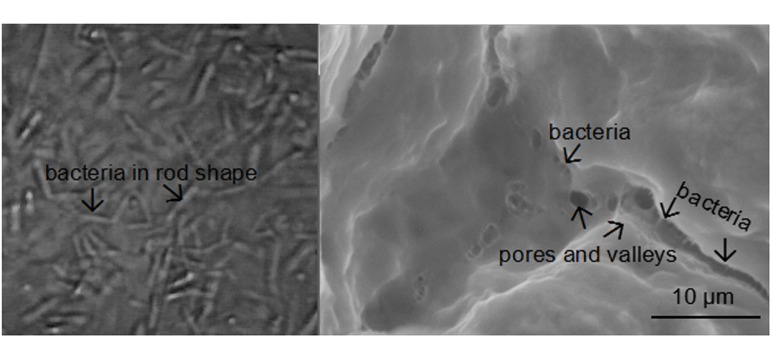
**(A)** Morphology of the bacteria; **(B)** morphology of the adsorbent. The bacteria is reflected by the tiny rod-shape structure with a similar size to it.

Fourier-transform infrared spectroscopy (FTIR) was performed to examine the interactions between bacteria and the adsorbents (**Figure 4**). Bands at 1420 and 1600 cm^-1^ are attributed to asymmetric and symmetric stretching vibrations of COO^-^ groups on the polymeric backbone of calcium alginate. Band at 1030 cm^-1^ is due to the C–O stretching of alcoholic groups of calcium alginate (Ye et al., 2016). There was not any significant change in peak position after bacteria were immobilized, indicating the adsorption mechanism was physical adsorption rather than chemical adsorption. SEM image confirmed that pore structure was successfully developed on surface of the adsorbents for bacteria adsorption. Many *Clostridium* species are rod shaped bacteria with a size of several micrometers (**Figure 5A**). The adsorbents contained an abundant amount of pore structure with a similar size to the bacteria (**Figure 5B**), under which condition the adsorption can be promoted and damaging exposure of bacteria to the extreme condition of high solvent concentration can be reduced.

The surface area and total pore volume are other factors affecting physical adsorption, and therefore studied to understand the structure-property relationships. The surface area reached 12.57 cm^2^/g, which was more than 10 times greater than sodium alginate that was used to prepare the adsorbents (1.02 cm^2^/g). Moreover, the adsorbent had total pore volume (0.016 cm^3^/g) several orders of magnitude greater than sodium alginate (0.00043 cm^3^/g). The increased pore volume and surface area were created by addition of cyclohexane as pore-forming agent, enhancing adsorption of the bacteria.

### Thermal Gravimetric Analysis of the Adsorbent

**FIGURE 6:**
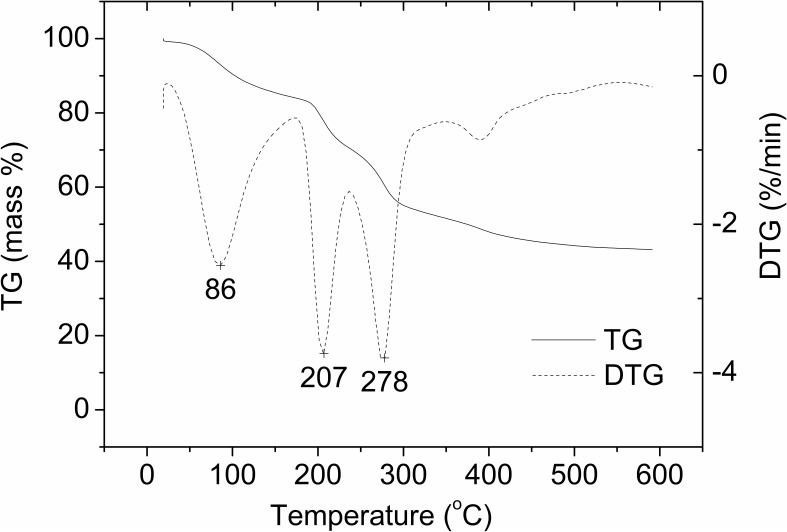
Thermal gravimetric analysis of the adsorbent.

**Figure 6** shows TG analysis of the adsorbent. The first negative peak at 86°C on the DTG curve is assigned to loss of free water and water linked through hydrogen bonds (Liu et al., 2016). The second mass loss corresponds to thermal decomposition of alginate with the fracture of glycosidic bonds and release of H_2_O (Liu et al., 2016), with a degradation peak centered at 207°C on the DTG curve. This result suggests the adsorbent should have high thermal stability at operating temperature of 37°C. The final step of mass loss with a peak at 278°C may be attributed to the carbonate formation.

### Comparison of Different Fermentations

**FIGURE 7:**
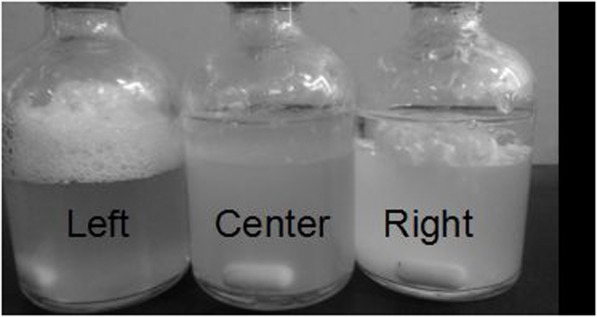
Comparison of different fermentations (Left: traditional fermentation without adsorbent or extractant; Center: fermentation with extractant but without adsorbent; Right: adsorbent assisted extractive fermentation).

When traditional fermentation (without either adsorbent or extractant) was carried out (**Figure 7**, Left), large amounts of bubbles were produced owing to production of CO_2_ and H_2_ by *C. acetobutylicum* with high activity (Jones and Woods, 1986). However, fermentation with extractant but without adsorbent yielded little gas products (**Figure 7**, Center), indicating the extractant presented strong toxicity to the *C. acetobutylicum*. On the other hand, bubbles were also released to the layer of extractant in adsorbent assisted ABE fermentation (**Figure 7**, Right) for the reason that adsorbent can improve solvent tolerance of bacteria under extremely high solvent concentration via reducing exposure of *C. acetobutylicum* to extractant. Our work first demonstrates successful *in situ* extraction of butanol using octanol as extractant.

### Product Analysis

**FIGURE 8:**
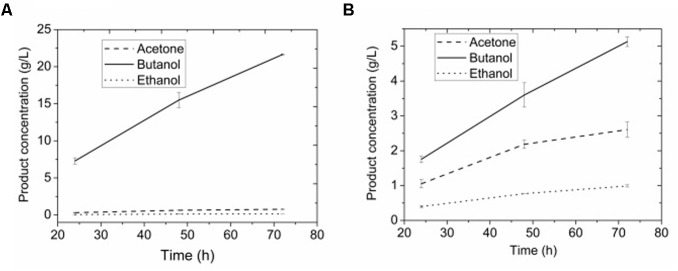
**(A)** Products in extractant layer by adsorbent assisted *in situ* extraction fermentation. **(B)** Products in broth by adsorbent assisted *in situ* extraction fermentation.

**Figures 8A,B** show the ABE products by adsorbent assisted ABE fermentation. 0.77 g/L acetone, 21.64 g/L butanol, and 0.16 g/L ethanol were presented in extracting layer while 2.61 g/L acetone, 5.13 g/L butanol, and 0.99 g/L ethanol were presented in fermenting broth after 3-day. The mean concentration of butanol was 9.25 g/L for the total volume (both aqueous and organic phases) of culture. The partition coefficients reached 4.22 for butanol at an aqueous phase/octanol volume ratio of 3:1. Meanwhile, octanol had partition coefficients of only 0.30 and 0.16 for acetone and ethanol, respectively, suggesting a high selectivity in extracting butanol.

**FIGURE 9:**
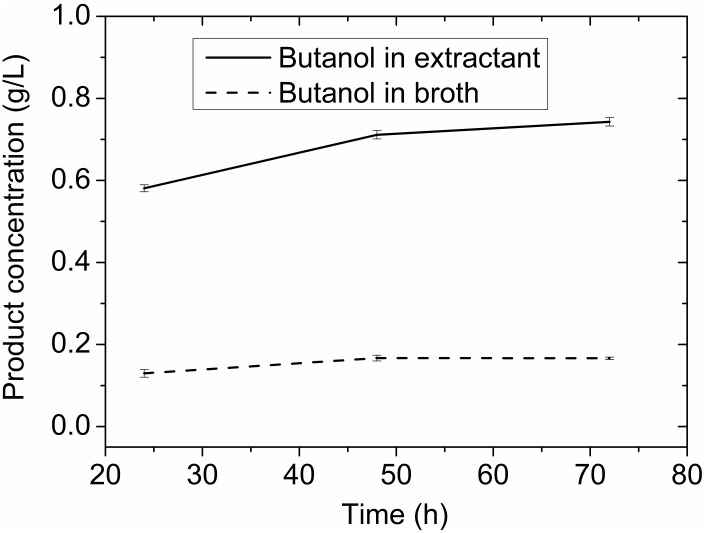
Butanol produced in fermentation with extractant but without adsorbent.

In a control experiment without adsorbent (**Figure 9**), little butanol was presented in either extractant layer or fermentation broth, which is consistent with toxic effect of octanol as reported (Groot et al., 1990). If the fermentation was carried out without either adsorbent or extractant, butanol concentration was 4.60 g/L. The butanol obtained (0.138 g) was only 37% of that (0.370 g) produced in the adsorbent assisted *in situ* extraction experiment. The extractive fermentation can increase butanol concentration in the extracting layer to more than 20 g/L together with the improved productivity, and reduce bacterial growth inhibition by decreasing toxic exposure to extremely high concentrations of octanol and solvent product, making the ABE fermentation more economic.

## Discussion

Commercial conversion of biomass to biobutanol has been severely limited by bottlenecks such as low productivity and high cost of product separation. In this paper, we report a technique using adsorbent (alginate) to promote biobutanol production by *C. acetobutylicum* under extreme condition of high concentration of octanol extractant. The strategy used here has the merit to improve total butanol production with respect to traditional culture approach by more than 2.5 fold. Additionally, this approach provided high butanol concentration in the octanol phase, without inhibition of bacterial growth by octanol-toxicity. The technique can simultaneously improve butanol production and reduce separation cost, with energy consumption reduced to ∼4–9 MJ/kg butanol using octanol extractant (Kurkijärvi et al., 2014).

The prepared adsorbent in this work has good affinity, high surface area for bacteria adsorption and good thermal stability, a matrix of properties desirable for potential industrial application. Though several studies have examined ABE fermentation with adsorbent, very little information is available for adsorbent assisted extractive fermentation. Huang et al. (2004) reported fibrous bed bioreactor immobilized cells and enhanced the yield of butanol by more than 68%, but their technique did not couple with extraction which case limited further increase of butanol. Levario et al. (2012) developed mesoporous carbon adsorbent for adsorption of butanol instead of the cells. Our work first demonstrated adsorbent assisted *in situ* extraction using octanol as extractant, which can make ABE fermentation more productive.

It is challenging to obtain high butanol concentration in ABE fermentation. Van Der Westhuizen et al. (1982) reported that vegetative cells of some *C. acetobutylicum* strain is very sensitive to butanol. The growth rate progressively decreased by concentrations of butanol between 4 and 8 g/L. It was in the toxic range for *C. acetobutylicum* with 4.60 g/L butanol from the traditional culture. The adsorbent assisted extractive fermentation provided butanol in extractant phase, without inhibition of bacterial growth by octanol and butanol toxicity. Therefore, much more butanol was produced with this new technique. Our result is consistent with previous report (Huang et al., 2004) that cell immobilization improved solvent tolerance of bacteria and allowed the cells to survive long in the solventogenic phase for long term solvent production.

The most effective extractant should have high partition coefficient of butanol. Biodiesel is a widely researched benchmark extractant for separating butanol from dilute ABE broth with a partition coefficient of butanol ∼1.5 (Yen and Wang, 2013). Our results demonstrated that *n-*octanol is far better than biodiesel for extractive fermentation. Moreover, *n-*octanol has a high selectivity of butanol over acetone and ethanol which are less profitable than butanol. Otherwise, the economics of the ABE fermentation will be severely impacted by continuous distillation with several distillers to separate butanol from acetone and ethanol.

Butanol fermentation is not economically viable mainly because the process requires high energy intensity of product separation (Kurkijärvi et al., 2014). Butanol has an energy density of only 36 MJ/kg. Energy requirement by distillation was 79.5 MJ/kg to obtain 99.9 wt% of *n-*butanol from 0.5 wt% broth (Matsumura et al., 1988). Gas stripping required energy of 13.8 MJ/kg (Kurkijärvi et al., 2014). It has been simulated that energy consumption can be reduced to ∼4–9 MJ/kg butanol if octanol is used as the extractant (Kurkijärvi et al., 2014). Compared with other alternative techniques, our extractive fermentation shows clear advantage in energy saving. Conventional extraction is limited to solvents which are biocompatible. However, the ability to use non-biocompatible, but effective solvent such as octanol offers a significant advantage, which is why our extractive fermentation can achieve a very low energy consumption.

Although extractive fermentation is an economic process for butanol recovery, the solvent used is another cost for this technique, besides that subsequent butanol recovery from extractant is also energy intensive due to the high boiling point of extractant. Octanol is relatively inexpensive compared to some benchmark extractant such as oleyl alcohol. With a low boiling point (287°C), Glyceryl tributyrate can save energy in subsequent separation of glyceryl tributyrate and butanol by distillation compared to oleyl alcohol (Anbarasan et al., 2012). Octanol has a boiling point of only193–195°C, and therefore can save more energy than glyceryl tributyrate and oleyl alcohol.

Energy consumption with this new technology (∼4–9 MJ/kg) accounts for up to 25% energy value of butanol. Cost of traditional substrates represents over 70% of total production cost, and can significantly influence the process economics. By coupling with fermentation using cheap macroalgae substrate, the technique developed here can potentially make ABE fermentation more economically practical in the future.

## Conclusion

Alginate adsorbent was successfully developed to assist *in situ* extraction of butanol from ABE fermentation. The adsorbent exhibited small particle size and high affinity for bacteria adsorption. Porous structure was successfully developed as demonstrated by SEM image. The adsorption was physical mechanism. 21.64 g/L butanol was presented in extracting layer compared to negligible products in fermentation with extractant but without adsorbent. Our work first demonstrated adsorbent assisted *in situ* extraction using octanol as extractant, which can improve solvent tolerance of bacteria under extremely high solvent concentration and make ABE fermentation more economically practical.

## Author Contributions

ZY and XH conceived the study. ZY and XC designed and wrote the manuscript. JS, EZ, and XS performed experiments. All authors reviewed the manuscript critically and approved the final version.

## Conflict of Interest Statement

The authors declare that the research was conducted in the absence of any commercial or financial relationships that could be construed as a potential conflict of interest.
